# Measuring the quality of life in hypertension according to Item Response Theory

**DOI:** 10.1590/S1518-8787.2017051006845

**Published:** 2017-04-24

**Authors:** José Wicto Pereira Borges, Thereza Maria Magalhães Moreira, Jeovani Schmitt, Dalton Francisco de Andrade, Pedro Alberto Barbetta, Ana Célia Caetano de Souza, Daniele Braz da Silva Lima, Irialda Saboia Carvalho

**Affiliations:** I Programa de Pós-Graduação em Saúde e Comunidade. Universidade Federal do Piauí. Floriano, PI, Brasil; II Programa de Pós-Graduação em Cuidados Clínicos em Enfermagem e Saúde. Programa de Pós-Graduação em Saúde Coletiva. Centro de Ciências da Saúde. Universidade Estadual do Ceará. Fortaleza, CE, Brasil; III Programa de Pós-Graduação em Engenharia de Produção. Universidade Federal de Santa Catarina. Florianópolis, SC, Brasil; IV Departamento de Informática e Estatística. Programa de Pós-Graduação em Engenharia de Produção. Universidade Federal de Santa Catarina. Florianópolis, SC, Brasil; V Unidade de Farmacologia Clínica. Núcleo de Pesquisa e Desenvolvimento de Medicamentos. Universidade Federal do Ceará. Fortaleza, CE, Brasil; VI Programa de Pós-Graduação em Saúde Coletiva. Universidade Estadual do Ceará. Fortaleza, CE, Brasil

**Keywords:** Quality of Life, Hypertension, Sickness Impact Profile, Surveys and Questionnaires, utilization, Validation Studies

## Abstract

**OBJECTIVE:**

To analyze the *Miniquestionário de Qualidade de Vida em Hipertensão Arterial* (MINICHAL – Mini-questionnaire of Quality of Life in Hypertension) using the Item Response Theory.

**METHODS:**

This is an analytical study conducted with 712 persons with hypertension treated in thirteen primary health care units of Fortaleza, State of Ceará, Brazil, in 2015. The steps of the analysis by the Item Response Theory were: evaluation of dimensionality, estimation of parameters of items, and construction of scale. The study of dimensionality was carried out on the polychoric correlation matrix and confirmatory factor analysis. To estimate the item parameters, we used the Gradual Response Model of Samejima. The analyses were conducted using the free software R with the aid of *psych* and *mirt*.

**RESULTS:**

The analysis has allowed the visualization of item parameters and their individual contributions in the measurement of the latent trait, generating more information and allowing the construction of a scale with an interpretative model that demonstrates the evolution of the worsening of the quality of life in five levels. Regarding the item parameters, the items related to the somatic state have had a good performance, as they have presented better power to discriminate individuals with worse quality of life. The items related to mental state have been those which contributed with less psychometric data in the MINICHAL.

**CONCLUSIONS:**

We conclude that the instrument is suitable for the identification of the worsening of the quality of life in hypertension. The analysis of the MINICHAL using the Item Response Theory has allowed us to identify new sides of this instrument that have not yet been addressed in previous studies.

## RESUMO

**OBJETIVO:**

Analisar o Miniquestionário de Qualidade de Vida em Hipertensão Arterial (MINICHAL) por meio da Teoria da Resposta ao Item.

**MÉTODOS:**

Estudo analítico realizado com 712 pessoas com hipertensão arterial atendidas em 13 unidades de atenção primária em saúde de Fortaleza, CE, em 2015. As etapas da análise pela Teoria da Resposta ao Item foram: avaliação da dimensionalidade, estimação dos parâmetros dos itens e construção da escala. O estudo da dimensionalidade foi realizado sobre a matriz de correlação policórica e análise fatorial confirmatória. Para a estimação dos parâmetros dos itens, foi utilizado o Modelo de Resposta Gradual de Samejima. As análises foram conduzidas no *software* livre R com o auxílio dos pacotes *psych* e *mirt*.

**RESULTADOS:**

A análise permitiu a visualização dos parâmetros dos itens e suas contribuições individuais na mensuração do traço latente, gerando mais informação, permitindo a construção de uma escala com um modelo interpretativo que demonstra a evolução da piora da qualidade de vida em cinco níveis. Quanto aos parâmetros dos itens, houve bom desempenho daqueles referentes ao estado somático, pois apresentaram melhor poder de discriminar os indivíduos com pior qualidade de vida. Os itens relacionados ao estado mental foram os que contribuíram com menor quantidade de informação psicométrica no MINICHAL.

**CONCLUSÕES:**

Conclui-se que o instrumento é indicado para a identificação da deterioração da qualidade de vida em hipertensão arterial. A análise do MINICHAL pela Teoria da Resposta ao Item permitiu identificar novas facetas desse instrumento ainda não abordadas em estudos anteriores.

## INTRODUCTION

The measure of health-related quality of life (HRQoL) is relevant for the investigation and evaluation of the health of individuals^5^. Commonly, the evaluation using a questionnaire follows a structure of similar questions, considering the various specific domains, including the physical, social, and psychological aspects and the spiritual factors^3,11,25^.

Because of its multifactorial characteristic, there are several HRQoL questionnaires, each bringing different models of quality of life (QOL) and equally differentiated implementation situations, always associated with health conditions or disease^3,11^. It is preferable to use a model clearly outlined for a specific context, rather than generic models^3,4^. Thus, the evaluation of the QOL is of particularly interest in patients with hypertension, since this condition has a significant epidemiological impact worldwide and it acts on the overall well-being of patients^1,4,25^. Such evaluation has become usual, among other ways, with the use of the *Miniquestionário de Qualidade de Vida em Hipertensão Arterial* (MINICHAL – Mini-questionnaire of Quality of Life in Hypertension).

This instrument has been developed in Spain (*mini-cuestionario de calidad de vida en la hipertensión arterial* [MINICHAL]) and validated by the classical test theory (CTT)^2^ to evaluate the impairment of the quality of life of persons with hypertension in their somatic and mental domains^23^. The MINICHAL has been translated, adapted transculturally, and validated for Brazil in 2008^19^ and, since then, it has been studied psychometrically^6,23,24^.

However, the properties evaluated, although relevant, have not been exhaustive and happened at the test level^2,6,23,24^. In the CTT, the analyses consider the behavior of the entire set of items that make up the instrument, characterizing analyzes at the test level. When using an instrument in clinical practice or in research, we need to have as many data as possible about its psychometric performance, strengths, and limitations, for the correct interpretation of the results obtained with the measure^23^.

Accordingly, the item response theory (IRT) has great power of knowledge of latent structures. Unlike CTT, which makes assumptions at the test level, IRT makes assumptions at the item level^1^. The IRT is considered a modern theory of psychometrics, as it focuses on each item of the measuring instrument and assumes that the performance of a particular test can be explained by individual characteristics, which are not directly observable, called latent traits^1,17^. Its growth has sophisticated theoretical background about the fundamentals of measurement in behavioral and social sciences, which must be studied and applied to improve the tests produced by CTT and formulate new tests^1^.

The analysis of the MINICHAL using IRT allows us to better evaluate its characteristics, enabling us to verify the latent trait underlying the instrument, observe what items are effectively measured, and position individuals and items on the same scale. With it, we can construct a scale with interpretable points in relation to the status of QOL of individuals, showing which behaviors require more effective health interventions. Thus, the objective of this study was to analyze the MINICHAL using the IRT.

## METHODS

This is an analytical study, carried out based on the item response theory to analyze the MINICHAL. This questionnaire, used in several studies in the world^14,16,22^ and Brazil^6,12,23,24^, contains seventeen items grouped into two domains. The domain “mental state” includes questions 1 to 9 and the domain “somatic manifestations” includes questions 10 to 16^20^. The original version in Spanish and the Brazilian version include an additional item, number 17, concerning the overall impact of hypertension in the QOL of the patient. This item, as it contains a general qualitative question, does not enter the sum of the scores in the CTT^20,22^.

Each item of the MINICHAL uses an adjectival scale with four possible answers (0 = no, absolutely; 1 = yes, a little; 2 = yes, enough; 3 = yes, a lot). Thus, using the CTT, the domain of mental status has a score between zero (best quality of life) and 27 (worst quality of life) points, while the domain of somatic manifestations has a score between zero (best quality of life) and 18 (worst quality of life) points. To show the general level of quality of life, the scores of mental status are added to the scores of somatic manifestations^6^.

The MINICHAL has been originally developed to be a self-administered questionnaire. However, in this study, because of the low educational level of the patients, the instrument was applied as a form. The instrument has suffered no modifications in its structure^19^. The items and their answers were read, and we requested the patient to choose an answer at the end of each item. We believe that this application had no negative impacts; on the contrary, it allowed the use of the instrument by persons with low schooling.

The study was carried out from January to October 2015, with persons with hypertension in the primary health care units (UAPS) of Fortaleza, State of Ceará, Brazil. The municipality has 105 primary health care units, distributed in six regional secretariats. We chose 13 units of primary health care using a random number table. For the draw, we considered the proportion of persons with hypertension in each region.

The sample consisted of 712 persons. Inclusion criteria were: being aged 18 years or more and registered as hypertensive in the primary health care units. We excluded patients with gestational hypertension or evident psychiatric illness.

The steps of the analysis of the instrument using the IRT were: evaluation of dimensionality, calibration or estimation of the parameters of the items, and construction of the scale. The study of dimensionality was carried out on the polychoric correlation matrix and we analyzed the principal components with a parallel analysis^9^. The polychoric correlations are suitable coefficients for items with ordinal scale. In addition, the dimensionality was also studied using the confirmatory factor analysis (CFA)^21^.

The parallel analysis with polychoric correlation matrix was performed with the aid of the *psych* ^a^ and the CFA with the *lavaan* ^b^, both from the free software R^c^. For the adjustment of the model in the CFA, we analyzed the following indexes: GFI (Goodness of Fit Index), AGFI (Adjusted Goodness of Fit Index), NFI (Normed Fit Index), TLI (Lucker-Lewis Index), CFI (Comparative Fit Index), and RMSEA (Root Mean Square Error of Approximation). As adjustment criteria, we adopted the following values: GFI greater than 0.90, AGFI greater than 0.90, NFI greater than 0.90, TLI greater than 0.90, CFI greater than 0.90, and RMSEA from 0.05 to 0.08^9^.

To estimate the parameters of the items, we used the gradual response model of Samejima, which assumes ordered response categories. The application of this model was carried out using the *Mirt* ^7^ of software R^c^. For this model, the probability *P*_i,k_ + (θ_j_) of a patient *j*, with latent trait (or level of quality of life) θ_j_, answering the item *i* in the category *j* or higher is given by:


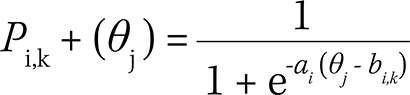


being *P*_i,0_ + (θ_j_) = 1, *P*_i,k_ + (θ_j_) = 0 and *k* the number of categories of item *i* (in this application *k* = four categories). The likelihood of response in the category k (k = 0, 1, ..., *k*) can be obtained by the difference of the probability in k and k+1. The term *a*_i_ is a parameter of item *i* that represents how much item *i* discriminates between different levels of the latent trait θ, and parameter *b*_i,k_ represents the position on the scale of the latent trait in which the category *k* of item *i* has a higher probability. In MINICHAL, the categories of response encoded by 0, 1, 2, and 3 are ordered such that *b*_i,0_ ≤ *b*_i,1_ ≤ *b*_i,2_ ≤ *b*_i,3_. It is desirable that the items have *a*_i_ > 0.7 and -3 < *b*_i,k_ < 3^1^. Items with values of *a* ≥ 1 are considered with reasonable power of discrimination^1^.

The construction of the scale was based on the anchor levels of items with good discrimination. Anchor levels are points in the scale selected by the analyst to be interpreted and anchor items are those selected for each anchor level^1^. For an item to be considered as anchor at a certain level of scale, we expect it to be answered positively for, at least, 65% of the patients with hypertension with this level of QOL, and by a proportion lower than 50% of patients with hypertension with an immediately below level of QOL. The difference in the proportion of patients with hypertension in these two levels should be, at least, 30%^1^. Because it is hard to satisfy all conditions^17^, the categories of items were positioned to achieve the proportion of 65% of response, called almost anchor levels.

In order to facilitate the discussion of the results, we performed a linear transformation of the scale of the latent trait (θ), usually set with an average of 0 (zero) and standard deviation of 1 (one), Scale (0:1), in the process to estimate the parameters of items and individuals. The scale was transformed into Scale (50:10), that is, with an average of 50 and standard deviation of 10.

The study was approved by the Research Ethics Committee of the *Universidade Estadual do Ceará* (Opinions 1.206.472 and 723.860).

## RESULTS

**Figure 1:**
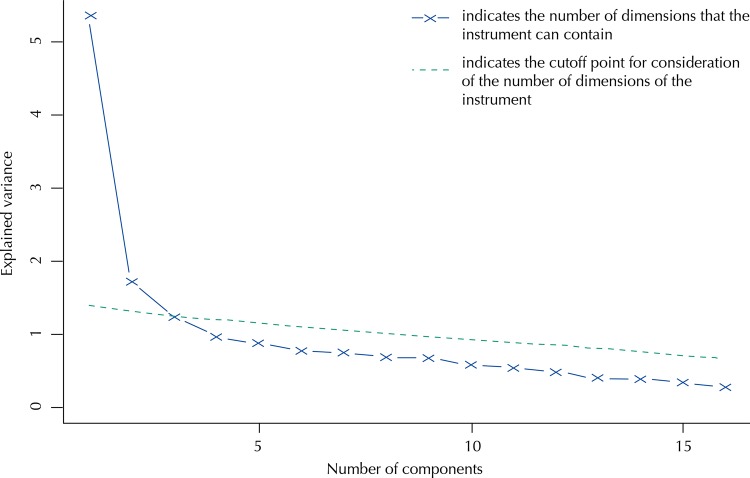
Principal component analysis and parallel analysis on the polychoric correlation matrix. Fortaleza, State of Ceará, Brazil, 2015.

The study of dimensionality using principal component analysis on the polychoric correlation matrix showed a dominant dimension, explaining 28.2% of the variance of responses of the items (the highest point in Figure 1). The presence of a dominant dimension indicates essential dimensionality, a sufficient condition for the application of one-dimensional IRT.

The presence of a second point above the dotted line, related to the parallel analysis, shows the presence of a second dimension. For the understanding of the one- and two-dimensional latent trait, we carried out a CFA. Table 1 presents the factor loadings, the estimates of the variance explained by factor, and the adjustment indexes of the CFA.

**Table 1:** Factor loadings and adjustment indexes of the confirmatory factor analysis (CFA) of the one and two-dimensional models of the MINICHAL. Fortaleza, State of Ceará, Brazil, 2015.

Items	Models		
	One-dimensional	Two-dimensional*	
	F1	F1	F2
1. Have you not been sleeping well?	0.47	0.40	0.12
2. Has it been difficult to keep your usual social relationships?	0.55	0.23	0.48
3. Has it been difficult to relate to people?	0.45	0.14	0.47
4. Do you feel that you are not exercising a useful role in life?	0.41	-0.05	0.66
5. Do you feel unable to make decisions and initiate new things?	0.46	-0.06	0.76
6. Have you been feeling constantly agonized and tense?	0.70	0.53	0.27
7. Do you feel that life is a continuous struggle?	0.38	0.20	0.27
8. Do you feel unable to enjoy your usual daily activities?	0.50	0.07	0.63
9. Have you been feeling exhausted and without strength?	0.75	0.61	0.24
10. Have you felt that you were sick?	0.62	0.55	0.13
11. Have you noticed difficulty breathing or shortness of breath without apparent cause?	0.67	0.77	-0.08
12. Have you had swollen ankles?	0.37	0.44	-0.07
13. Have you noticed that you urinate more frequently?	0.25	0.20	0.08
14. Have you been feeling your mouth dry?	0.56	0.50	0.12
15. Have you been feeling chest pain without making a physical effort?	0.67	0.79	-0.10
16. Have you noticed numbness or tingling in any part of your body?	0.53	0.63	-0.09
17. Would you say your hypertension and its treatment have affected your quality of life?	0.42	0.33	0.15
Explained variance (%)	28.20	28.20	9.33

CFA adjustment indexes for each model			
GFI	0.973	0.985	
AGFI	0.956	0.976	
NFI	0.926	0.959	
TLI	0.934	0.972	
CFI	0.943	0.976	
RMSEA	0.067	0.043	

GFI: Goodness of Fit Index; AGFI: Adjusted Goodness of Fit Index; NFI: Normed Fit Index; TLI: Lucker-Lewis Index; CFI: Comparative Fit Index; RMSEA: Root Mean Square Error of Approximation

* Includes oblimin rotation; correlation between factors 1 and 2 was 0.441.

Bold values represent the highest factor loading and the item loading in one of the factors.

In the two-dimensional model, ten items presented high factor loadings in the first factor (F1, items: 1, 6, 9, 10, 11, 12, 14, 15, 16, and 17) and another six items in the second factor (F2, items: 2, 3, 4, 5, 7, and 8). The items of dimension 1 (F1) are related to the factors of the somatic state of the patient, while items in dimension 2 (F2) are related to the mental state of the patient. Three items (1, 6, and 9) were linked in a different dimension of the study of validation of the MINICHAL for Brazil (Table 1, two-dimensional model).

According to Table 1, we can observe that in the adjustment of the one- and two-dimensional models, only item 13 presented a loading lower than 0.30, indicating that it is not well related to the latent trait of “quality of life in hypertension”. In turn, item 17 (“Would you say your hypertension and its treatment have affected your quality of life?”) was linked to the domain of somatic manifestations.

The dimensional models show two perspectives to consider the latent trait in question: a one-dimensional perspective and another two-dimensional one, both with criterion of validity shown by the CFA, as the indexes of adjustment of the models remained within the criteria adopted. In addition, the two factors that make up the two-dimensional model have a correlation of 0.441 (Table 1). These facts define the construct.

In this work, the construction of the scale was based on the one-dimensional model, which, as discussed earlier, is justified because of the presence of a dominant dimension. In addition, the set of items generally measures a single factor, as expressed by the indexes of adjustment of the CFA of the one-dimensional model (Table 1). Additionally, in the MINICHAL, when applied by the CTT, the scores of the mental status are added to the scores of the somatic manifestations to express the general level of quality of life, for the processing of the data in a one-dimensional approach.

Table 2 presents the estimates of the parameters of the items of the one-dimensional IRT model. The items that best discriminated the patients with hypertension with poor quality of life were 2, 6, 9, 10, 11, 14, 15, and 16, which have a higher discrimination parameter (*a*). Other items presented values of *a* next to the reference adopted (*a* > 0.70). In this regard, items 10, 11, 14, 15, and 16 of the somatic state have better power to discriminate individuals with worse QOL than the items that measure the mental state and which had, in most of them, parameters *a* next to the reference adopted.

**Table 2:** Parameters of the items on the scale (0.1) of the MINICHAL. Fortaleza, State of Ceará, Brazil, 2015.

Items^a^		Parameters			
		a (SE)	b2 (SE)	b3 (SE)	b4 (SE)
Mental state					
1	Have you not been sleeping well?	0.90 (0.11)	-0.17 (0.09)	0.67 (0.10)	1.95 (0.12)
2	Has it been difficult to keep your usual social relationships?	1.12^b^ (0.15)	1.30 (0.13)	2.06 (0.17)	2.76 (0.21)
3	Has it been difficult to relate to people?	0.87 (0.15)	2.03 (0.14)	2.95 (0.19)	3.93 (0.26)
4	Do you feel that you are not exercising a useful role in life?	0.76 (0.12)	1.33 (0.11)	2.23 (0.13)	3.17 (0.15)
5	Do you feel unable to make decisions and initiate new things?	0.88 (0.12)	1.23 (0.11)	2.09 (0.13)	3.17 (0.18)
6	Have you been feeling constantly agonized and tense?	1.66^b^ (0.14)	-0.37 (0.12)	0.52 (0.12)	1.25 (0.15)
7	Do you feel that life is a continuous struggle?	0.70 (0.09)	-2.19 (0.11)	-0.82 (0.09)	0.73 (0.09)
8	Do you feel unable to enjoy your daily activities?	0.97 (0.12)	0.77 (0.10)	1.69 (0.12)	2.79 (0.16)
9	Have you been feeling exhausted and without strength?	1.95^b^ (0.17)	-0.29 (0.13)	0.54 (0.14)	1.28 (0.19)
Somatic manifestations					
10	Have you felt that you were sick?	1.34^b^ (0.12)	-0.25 (0.11)	0.81 (0.12)	1.67 (0.15)
11	Have you noticed difficulty breathing or shortness of breath without apparent cause?	1.53^b^ (0.16)	0.57 (0.12)	1.46 (0.17)	2.20 (0.23)
12	Have you had swollen ankles?	0.68 (0.10)	0.90 (0.09)	2.26 (0.11)	3.25 (0.14)
14	Have you been feeling your mouth dry?	1.15^b^ (0.12)	0.15 (0.10)	1.09 (0.12)	1.75 (0.13)
15	Have you been feeling chest pain without making a physical effort?	1.52^b^ (0.16)	0.64 (0.12)	1.68 (0.19)	2.57 (0.30)
16	Have you noticed numbness or tingling in any part of your body?	1.06^b^ (0.11)	-0.38 (0.10)	1.14 (0.11)	1.99 (0.14)
17	Would you say your hypertension and its treatment have affected your quality of life?	0.78 (0.10)	0.83 (0.10)	1.75 (0.11)	2.85 (0.14)

a: parameter of discrimination; b: parameter of difficulty or positioning; SE: standard error of the parameter

^a^ Item 13 was removed from the estimation of the parameters because it presented low parameter of discrimination.

^b^ Items with better power of discrimination.

However, item 13 (*a* = 0.45) was below the criterion adopted, indicating little discrimination in terms of the quality of life in hypertension. This item already had shown weakness in the analysis of dimensionality. Given that, it was disregarded in the construction of the interpretation of the scale (Table 2).

The items with lower response in the higher categories (categories 2 or 3) were those related to the difficulty in relating to persons, the feeling that they are not exercising a useful role in life, being unable to make decisions and initiate new things, and swelling in the ankles. This is due to their high values for parameters *b*, in which the most negative answers to the QOL were given only by the patients with hypertension with poor quality of life in hypertension. Parameters *b* indicate the position in the scale in which the item had more information (Table 2).

**Figure 2:**
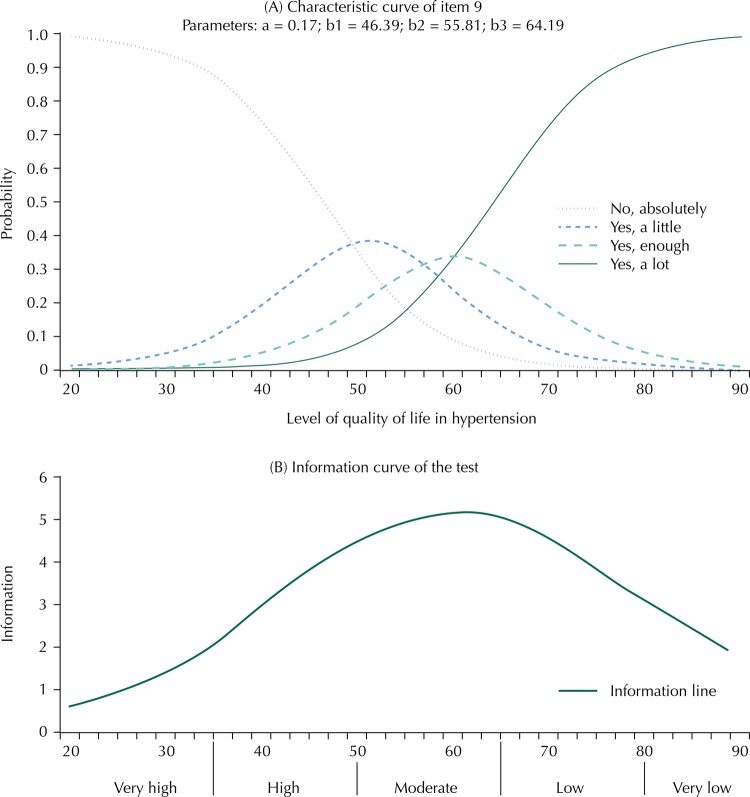
Characteristic curve of the item (A) and information curve (B) of MINICHAL in a 50:10 scale. Fortaleza, State of Ceará, Brazil, 2015.

Exemplifying the item response process and the amount of data that the MINICHAL showed when analyzed by the IRT, Figure 2 shows (a) characteristic curve of item 9 and (b) test information curve.

From the characteristic curve of item 9 – “Have you been feeling exhausted and without strength?” (Figure 2, A), we can interpret that patients with a HRQoL level less than 46.39 points in the scale will probably choose the category “no, absolutely”. On the other hand, persons with level between 46.39 and 55.81, will probably choose the category “yes, a little”. Persons with level between 55.81 and 64.19 points will probably choose the category “yes, enough” and the persons with HRQOL above 64.19 will probably choose the category “yes, a lot”. Figure 2 (B) shows the test information curve in which the instrument has more information (higher curve) in the range from 40 to 80 points. This means that the instrument is best suited to measure the level of quality of life in hypertension in patients who are in this range, which represents lower QOL in hypertension.

Table 3 presents the levels of the scale and their interpretation. Persons under treatment for hypertension with score < 35 had very high quality of life in hypertension. At the next level, and reaching the median of the scale, there was increased feeling that life is a continuous struggle. At level 50 to 65, descriptors of the somatic domain predominated, and at the next level (65 to 80) the descriptors of the mental domain predominated, indicating that the deterioration of the QOL in hypertension begins with somatic manifestations and deepens with mental manifestations. We can observe that more than half of the patients were located in the first two levels of the scale, representing high quality of life inhypertension.

**Table 3:** Interpretation of the levels of the scale proposed for the MINICHAL. Fortaleza, State of Ceará, Brazil, 2015.

Anchoring of items	Level	Description	% of patients
Category “No, absolutely”	Below 35 (Very high quality of life in hypertension)	Patients at this level of the scale have very high quality of life in hypertension.	5.80
I7b2	35 to 50 (High quality of life in hypertension)	In general, patients located at this level still have a high quality of life in hypertension; however, they have a slight sensation that life is a continuous struggle.	45.80
I1b2, I6b2, I9b2, I10b2, I11b2, I14b2, I15b2, I16b2, I6b3, I7b3, I9b3	50 to 65 (Moderate quality of life in hypertension)	Patients at this level have moderate quality of life, have an enough sensation that life is a continuous struggle and, in general, feel a little sick. Although in a lighter degree, they complain of not sleeping well, feeling tense, agonized, exhausted, and without strength. Other somatic manifestations, such as difficulty breathing, dry mouth, numbness and tingling of some part of the body, and chest pain without making an effort, are more likely to be present in patients located at this level of quality of life in hypertension.	40.00
I2b2, I3b2, I4b2, I5b2, I8b2, I12b2, I1b3, I2b3, I5b3, I8b3, I10b3, I11b3, I14b3, I15b3, I16b3, I17b3, I1b4, I6b4, I7b4, I9b4, I10b4, I11b4, I14b4, I16b4	65 to 80 (Low quality of life in hypertension)	At this level, there is a low quality of life. The hypertension and the treatment considerably affect the quality of life. There is a worsening of the sleep quality and issues related to mental health, such as: difficulty in relating to people, feeling worthlessness, inability to make decisions and initiate new things, and enjoying usual daily activities. There is a worsening of the somatic symptoms described at the previous level (chest pain, tingling, exhaustion) in addition to the appearance of swelling in the ankles.	8.10
I2b3, I4b3, I12b3, I2b4, I4b4, I5b4, I8b4, I12b4, I15b4, I17b4	Above 80 (Very low quality of life in hypertension)	Patients at this level of the scale have very low quality of life. The hypertension and the treatment considerably affect the quality of life, and there is a worsening of all mental and somatic manifestations observed in the instrument.	0.30

I: item; b2: category “yes, a little”; b3: category “yes, enough”; b4: category “yes, a lot”

## DISCUSSION

The analysis of the MINICHAL using the IRT provided additional data in terms of validity and interpretation.

From the study of dimensionality, we confirmed that the MINICHAL measures the quality of life in the somatic and mental aspects^6,12,20,23,24^. We also observed good fit in a one-dimensional model, showing the possible treatment of the MINICHAL in the one and bi-dimensional approach^5^. However, aiming at the construction of a relatively simple interpretative scale of the MINICHAL, we chose to use the one-dimensional approach^15,19^. For the interpretation of the items, we used their representation in the somatic dimensions and mental state, in order to characterize the explanation in those dimensions.

On the other hand, we highlight that the adjustment of a scale with two factors would be possible with the use of the Multidimensional Item Response Theory (MIRT), which would allow the study of the parameters of items in their respective dimensions. However, because of the complexity of a two-dimensional scale, we chose to use the one-dimensional model of the IRT.

The placement of items and individuals in a multidimensional space is still an incipient subject. Many proposals have emerged for analysis and modeling with the IRT, but we still do not have an established methodology as in classical theory or in one-dimensional IRT^5^.

The analysis of dimensionality of the MINICHAL showed small differences when compared to the Brazilian validation study^20^. Items 1 (“Have you not been sleeping well?”), 6 (“Have you been feeling constantly agonized and tense?”), and 9 (“Have you been feeling exhausted and without strength?”) showed high factor loadings in the dimension of somatic manifestations. In the validation study, these items showed correlation with the dimension of mental state^20^. This difference came from the analysis of dimensionality performed in the Brazilian validation study, which has used an analytical technique that assumes the responses of the items in a continuous scale. In this work, we considered an approach suitable for ordinal data (polychoric correlations)^21^.

Theoretically, the moderate correlations of items 1, 6, and 9 in the somatic dimension can be explored as follows: item 1) changes in sleep can lead to changes in the physical, occupational, cognitive, and social functioning of the person with hypertension, compromising the quality of life and being manifested as fatigue symptoms^10,13^; item 6) this item has two commands (agonized and tense) in one question, and the command designated by tension is connected to somatic changes and, thus, directed the item to the correlation with this dimension; item 9) it has the commands exhausted and without strength, understood by the participants of the research as elements of physical, and not mental, exhaustion.

On the other hand, item 13 (“Have you noticed that you urinate more frequently?”) is not linked to any of the dimensional models tested. In the reality studied, we assumed that the affirmative response of patient to this item may not be related to the treatment of hypertension with the use of diuretics, as the increased frequency to urinate can be a result of other causes, not being part of the latent trait measured by the MINICHAL.

Regarding the parameters of the items, the items related to the somatic state had a good performance, as they have presented better power to discriminate individuals with worse QOL. Discrimination is an important psychometric property for the item to differentiate patients in the levels of QOL in hypertension^8^.

Of the items of the mental state, the best performance was related to the difficulty in maintaining social relations. Moreover, the items related to mental state were those which contributed with less psychometric data in the MINICHAL, especially item 12 (“Have you had swollen ankles?”), which has low discrimination. The amount of information of the item indicates the precision of the measurement associated with each level of the scale^18^. An alternative for this result is the addition of well formulated items about the mental state to increase the amount of information and better discriminate individuals in the aspects of this dimension.

Parameter *b,* because it is measured in the same scale of the latent trait, provides the place for the positioning of the item in the scale. This characteristic enables us to understand the meaning of the score, and not only as an indicator of QOL, as presented by the CTT^17^. Its values should be between -5 and 5 as a way to allow the measurement of the QOL on its good and bad aspects^1^. The results of the MINICHAL demonstrate most positive parameters of difficulty. This shows, in interpretative terms, an instrument that measures the poor QOL in hypertension. Thus, there is the need to formulate items with information on very high quality of life, filling that gap in the instrument.

According to the scale built, we can notice the evolution in the worsening of the QOL in five levels. In the first level, despite representing those with very high quality of life, the instrument did not provide additional data that qualified this level. The following levels qualitatively showed the worsening points of the QOL that start with symptoms related to the somatic factor, followed by the impairment of the mental state. This interpretation of the levels is a property of IRT models, which enables the creation of a care plan for the patient according to the individual score^17^. Thus, the instrument is suitable for the identification of worsening of the QOL in hypertension.

The analysis of the MINICHAL using the IRT allowed us to identify new sides, such as the organization of items in a scale, the composition of interpretation for the levels, and the qualification of these levels. In the scale proposed, feeling that life is a continuous struggle demarcates the beginning of the worsening of the QOL with problems related to somatic manifestations followed by problems of the mental state.

In relation to limitations, the MINICHAL did not provide information that qualified persons with very high quality of life. In addition, there is the need to create a digital platform so that it can be used in future research studies.

The construction of an interpretation for each level of the scale seems to fill the gap in studies that measure health behavior, which goes beyond the responses commonly provided by instruments of quality of life.

## Footnotes

a Revelle W. Psych: procedures for psychological, psychometric, and personality research. Evaston: Northwestern University; 2015.

b Rosseel Y. Lavaan: Latent Variable Analysis. Version 0.5-20. Gent: 2015 [cited 2017 Feb 18]. Available from: http://lavaan.org

c R Development Core Team. R: a language and environment for statistical computing. Version 2.13.2. Vienna: R Foundation for Statistical Computing; 2011 [cited 2017 Feb 18]. Available from: https://cran.r-project.org/bin/windows/base/old/2.13.2/
